# Resilience Among Displaced and Non-Displaced Ukrainian Women During the War: An Exploratory Cluster Analysis

**DOI:** 10.3390/bs16060988

**Published:** 2026-06-15

**Authors:** Alexis Cloquell-Lozano, Carmen Moret-Tatay, Carlos Novella-García, Iryna Zharova

**Affiliations:** 1 Faculty of Teacher Training and Educational Sciences, Universidad Católica de Valencia San Vicente Mártir, 46001 Valencia, Spain; alex.cloquell@ucv.es; 2 Faculty of Psychology, Universidad Católica de Valencia San Vicente Mártir, 46001 Valencia, Spain; mariacarmen.moret@ucv.es; 3 Department of Therapy and Rehabilitation, National University of Ukraine on Physical Education and Sport, 03150 Kiev, Ukraine; aniri2002@ukr.net

**Keywords:** displaced women under temporary protection, women, resilience, emotional experiences, war

## Abstract

Russia’s invasion of Ukraine has exposed millions of individuals to traumatic experiences, displaced them under temporary protection, and caused psychological distress. This exploratory study examined resilience, emotional experiences, and psychosocial profiles among displaced and non-displaced Ukrainian women affected by the war. A total of 249 adult women participated, including 122 displaced women under temporary protection residing in Spain and 127 women living in Ukraine. Participants completed the Brief Resilient Coping Scale (BRCS) and the Scale of Positive and Negative Experience (SPANE). Group comparisons and cluster analyses were conducted to identify distinct psychosocial patterns. Displaced women showed slightly higher resilience scores than non-displaced women, although differences were not statistically significant. Non-displaced women reported significantly higher levels of both positive and negative emotional experiences, suggesting greater emotional intensity among those remaining in Ukraine. Cluster analyses identified three psychosocial profiles: an adaptive profile characterized by high positive affect, low negative affect, stronger social support, and higher resilience; a vulnerable profile marked by low social support, elevated negative affect, and lower resilience; and an intermediate profile showing high negative affect despite moderate-to-high social support. Although displaced women under temporary protection were more represented in the vulnerable profile, this association was not statistically significant. The findings highlight the heterogeneity of psychological adaptation during war and displacement and emphasize the protective role of resilience and social support.

## 1. Introduction

Russia’s invasion of Ukraine (2022) has resulted in casualties, population displacement, and ongoing political tensions, leaving millions of individuals exposed to traumatic experiences and psychological vulnerability. Living in a war zone not only represents a violation of fundamental human rights but also affects the present and long-term physical and mental well-being of individuals and societies (Singhal, 2019). Exposure to war-related trauma has been associated with post-traumatic stress disorder (PTSD), anxiety, depression, hopelessness, and suicidal ideation (Steel et al., 2009; Barzilay et al., 2021; Bryan et al., 2015; Kovess-Masfety et al., 2021; Maley, 2011). In addition, displaced under temporary protection, disruption of social networks, loss of livelihoods, and limited access to services may intensify psychological distress and reduce coping capacity (Jankovic et al., 2013).

By the end of 2023, the war in Ukraine had caused the forced displacement of approximately 11 million people, including 6.3 million refugees and 3.7 million internally displaced persons (UNHCR, 2026). In response to this humanitarian crisis, the European Union activated Council Directive 2001/55/CE, granting temporary protection and access to housing, healthcare, employment, and social assistance to displaced Ukrainians. Spain has become one of the European countries receiving the largest number of Ukrainian refugees, with more than 170,000 temporary protection permits granted. According to the report “Lives on Hold”: Profiles and Intentions of Refugees from Ukraine (UNHCR, 2022), women and children account for 87% of refugee family members. In Spain, women represent approximately 63% of temporary protection beneficiaries. These figures highlight the importance of examining the experiences of Ukrainian women affected by war and displacement.

Recent updates to the International Classification of Diseases (ICD-11) have formally recognized Complex Posttraumatic Stress Disorder (CPTSD) and Prolonged Grief Disorder (PGD), disorders that may be particularly relevant among populations exposed to prolonged and repeated traumatic events (Shevlin et al., 2022). Previous studies have shown that PTSD frequently coexists with depression, anxiety disorders, alcohol misuse, and psychosomatic symptoms among conflict-affected populations (Shalev et al., 2019; Aloni & Ben-Ari, 2024). Women exposed to traumatic events appear especially vulnerable, presenting higher probabilities of developing PTSD and related psychological difficulties (Shalev et al., 2019; Aloni & Ben-Ari, 2024). Both active exposure to armed conflict and indirect exposure through displacement, insecurity, and loss may contribute to persistent emotional distress among Ukrainian civilians.

Research conducted with displaced and non-displaced Ukrainians has also revealed important differences in psychological and physical symptoms. Internally displaced women have reported lower levels of pain symptoms than externally displaced and non-displaced women, whereas externally displaced individuals have shown higher levels of insomnia (Vorobiova & Schilling, 2023). Lower physical activity levels have been associated with greater PTSD symptoms, depression, pain, and insomnia, while subjective perceptions of illness have shown positive associations with psychological distress (Vorobiova & Schilling, 2023). These findings suggest that psychological outcomes among Ukrainian women may vary according to displacement experiences as well as broader sociodemographic and contextual factors.

In this context, resilience has emerged as a relevant construct for understanding psychological adaptation following adversity. Resilience refers to the ability to adapt positively and recover from stressful or traumatic experiences (Southwick et al., 2014; Solà-Sales et al., 2021; Murphy et al., 2021). Higher levels of resilience have been associated with greater psychological well-being, optimism, and hope, together with lower levels of anxiety and depression (Bonanno, 2004; Moret-Tatay & Murphy, 2022). Studies of displaced under temporary protection populations indicate that resilience may buffer against PTSD and other psychological symptoms following exposure to trauma and prolonged stress (Wingo et al., 2010; Rutter, 2007; Luthar et al., 2000). At the same time, resilience is influenced by social and cultural factors, including coping strategies, social support, belonging, and identity processes (Miller & Rasmussen, 2010). For displaced populations, experiences of migration and separation from familiar environments may shape both emotional experiences and resilience processes, making these factors particularly relevant in the Ukrainian context.

Given these considerations, the present exploratory study aimed to examine resilience, emotional experiences, and psychosocial functioning among Ukrainian women affected by the war, comparing displaced and non-displaced participants. Specifically, the study sought to: (1) analyze differences in resilience and positive and negative emotional experiences according to displacement status, and (2) identify distinct psychosocial profiles based on emotional experiences and perceived social support. To address these objectives, cluster analyses were conducted using emotional and social support variables, followed by comparisons of resilience (BRCS) and displacement status across the identified profiles. Given the exploratory nature of the study, no directional hypotheses were established. The findings may contribute to a better understanding of psychological adaptation processes among Ukrainian women affected by war and displacement and may help inform future psychosocial support interventions.

## 2. Materials and Methods

### 2.1. Participants and Procedure

The study involved a sample of 249 adult women (aged 18 years or older), comprising 122 Ukrainian displaced women residing in Spain under temporary protection and 127 women residing in Ukraine, who were selected through convenience sampling and voluntarily participated. In accordance with Council Directive 2001/55/EC, participants residing in Spain were beneficiaries of temporary protection. For consistency, they are referred to throughout the manuscript as women under temporary protection rather than refugees. They were recruited through convenience sampling through social networks, community organizations, and support groups assisting Ukrainian populations. Participation was voluntary and anonymous, and all participants provided informed consent prior to completing the study. In terms of marital status, 53.8% were single, 40.2% were married, and 6% were separated. Regarding their educational background, 7.6% had completed primary education, 47.8% had secondary education, and 44.6% had higher education. All measures were self-reported, and no missing values were present in the dataset. All participants provided written informed consent, and the study received approval from the Research Ethics Committee of the institution (UCV/2022-2023/026). This study forms part of a broader research project examining the psychological and psychosocial consequences of the war in Ukraine; however, the present work addresses distinct objectives by specifically focusing on resilience, emotional experiences, and psychosocial profiles among displaced and non-displaced Ukrainian women (Moret-Tatay et al., 2025).

### 2.2. Instruments

Following the administration of a sociographic questionnaire, an adapted version of the Resilience and Social Support questionnaire was utilized. Additionally, the Ukrainian version of the Positive and Negative Experience scale was employed. First, the Brief Resilient Coping Scale (BRCS), developed by Sinclair and Wallston (2004), was chosen to measure resilience. This tool assesses optimism, perseverance, creativity, and positive growth in challenging situations, defining resilient coping as an active problem-solving approach. The BRCS has demonstrated adequate reliability and validity for assessing resilience. The original scale consists of four items representing a single dimension, and it showed acceptable internal consistency (ω = 0.795).

The Multidimensional Scale of Perceived Social Support (MSPSS) (Zimet et al., 1988) was included as a measure of a 12-item scale to evaluate perceived social support from three sources: Family, Friends, and Significant Others. Responses are provided on a seven-point Likert scale, yielding total scores ranging from 12 to 84, with higher scores reflecting greater perceived social support. It showed an excellent reliability (ω = 0.935).

Lastly, the Scale of Positive and Negative Experiences (SPANE) includes two subscales: positive experiences (SPANE-P) and negative experiences (SPANE-N), each containing six items that measure three general and three specific emotions. It uses a five-point Likert scale ranging from 1 (“very rarely or never”) to 5 (“very often or constantly”). Previous studies have shown that the SPANE possesses optimal psychometric properties within the Ukrainian population (Moret-Tatay et al., 2025; Morimitsu & Akerkar, 2023). The positive subscale (SPANE-P) demonstrated excellent reliability (ω = 0.910), while the negative subscale (SPANE-N) showed good reliability (ω = 0.831).

### 2.3. Data Analysis

To identify subgroups of women with distinct emotional and social support profiles, a cluster analysis was performed using positive and negative affect (SPANE) and th MSPSS subscales assessing support from Significant Others, Family, and Friends. Prior to clustering, all variables were standardized (z-scores; M = 0, SD = 1) to prevent scale differences from distorting distance-based computations. K-means clustering was applied iteratively for k = 2 through k = 7. The optimal number of clusters was evaluated using two criteria simultaneously: (a) the elbow method, based on within-cluster sum of squares (inertia), and (b) the silhouette coefficient, which quantifies cluster cohesion and separation. Based on these criteria, a three-cluster solution (k = 3; silhouette = 0.245) was selected as offering the best balance between statistical fit and psychological interpretability. The silhouette coefficient (0.245; k = 3) indicated modest cluster separation, a finding that is common in psychological research where constructs often overlap and group boundarieare not sharply defined. The cluster analysis successfully classified all subjects into one of the identified clusters, with no unassigned cases. Statistically significant differences across profiles on each of the five clustering variables were confirmed using Kruskal–Wallis tests. All analyses were conducted in Python (v3.x) using scikit-learn and scipy. To further characterize the profiles, two additional variables were examined: (1) BRCS scores, and (2) displacement status (not-displaced vs. displaced under temporary protection). Group differences in resilience across profiles were tested using a Kruskal–Wallis test followed by Mann–Whitney U pairwise post hoc tests with Bonferroni correction (adjusted α = 0.017). The association between cluster membership and displacement status was assessed via a Pearson chi-square test, with Cramér’s V as the effect size indicator.

## 3. Results

The results from Table 1 provide a description of the scores for displaced and non-displaced individuals.

The Brief Resilient Coping Scale (BRCS) scores indicate that displaced individuals have a slightly higher mean score (M = 15.07, SD = 3.14) compared to non-displaced individuals (M = 14.36, SD = 3.24), suggesting better coping abilities among the displaced, although this difference is not statistically significant (*p* = 0.08). In terms of positive experiences, measured by the Scale of Positive and Negative Experience Positive (SPANE_P), non-displaced individuals report significantly higher positive experiences (M = 21.13, SD = 4.34) than displaced individuals (M = 19.87, SD = 4.17), with a *p*-value of 0.02. Conversely, negative experiences, as measured by the Scale of Positive and Negative Experience Negative (SPANE_N), are significantly higher among non-displaced individuals (M = 17.91, SD = 4.05) compared to displaced individuals (M = 16.13, SD = 3.96), with a highly significant *p*-value of 0.001.

Lastly, three multiple linear regression analyses were conducted. Age and group status were entered as independent variables in each model. Group status was dummy-coded as 0 = No and 1 = Yes. Separate models were estimated for resilience BRCS_T, positive affect SPANE_P, and negative affect SPANE_N.

For resilience, the overall regression model was not statistically significant, F(2, 246) = 1.58, *p* = 0.208, and explained a very small proportion of variance in BRCS_T, R^2^ = 0.013, adjusted R^2^ = 0.005. Group status was not a significant predictor of resilience after adjusting for age, B = 0.62, SE = 0.46, β = 0.10, t = 1.33, *p* = 0.185, 95% CI [−0.30, 1.53]. Age was also not significantly associated with resilience, B = 0.01, SE = 0.02, β = 0.03, t = 0.38, *p* = 0.701, 95% CI [−0.03, 0.04]. Thus, among women, neither age nor group status significantly predicted resilience.

For positive affect, the regression model was statistically significant, F(2, 246) = 3.73, *p* = 0.025, accounting for approximately 3.0% of the variance in SPANE_P, R^2^ = 0.030, adjusted R^2^ = 0.022. However, neither individual predictor reached statistical significance. Group status was not significantly associated with positive affect after controlling for age, B = −0.83, SE = 0.62, β = −0.10, t = −1.35, *p* = 0.178, 95% CI [−2.05, 0.38]. Age was also not a significant predictor, although the coefficient was negative, B = −0.03, SE = 0.02, β = −0.10, t = −1.42, *p* = 0.157, 95% CI [−0.08, 0.01].

For negative affect, the regression model was statistically significant, F(2, 246) = 11.30, *p* < 0.001, explaining 8.4% of the variance in SPANE_N, R^2^ = 0.084, adjusted R^2^ = 0.077. Age was a significant negative predictor of negative affect, B = −0.07, SE = 0.02, β = −0.22, t = −3.15, *p* = 0.002, 95% CI [−0.12, −0.03]. This indicates that older women reported lower levels of negative affect. Group status was not a significant predictor of negative affect after adjustment for age, B = −0.90, SE = 0.57, β = −0.11, t = −1.58, *p* = 0.116, 95% CI [−2.02, 0.22].

### 3.1. Profile Characteristics

The three-cluster solution yielded the following profile sizes: Profile A (n = 69, 27.7%), Profile B (n = 104, 41.8%), and Profile C (n = 76, 30.5%). Kruskal–Wallis tests showed statistically significant differences between profiles across all five clustering variables, with all test statistics exceeding H = 93 and all *p* values below 0.001. These results indicate that the three profiles were clearly differentiated in terms of positive affect, negative affect, and perceived social support.

Profile B, labelled High Positive Affect/Low Negative Affect/Moderate-to-High Social Support, was characterized by the highest level of positive affect (M = 23.85), the lowest level of negative affect (M = 14.28), and relatively high perceived social support across the three MSPSS subscales: Significant Other (M = 6.23), Family (M = 6.13), and Friends (M = 6.04). This profile therefore represents the most favourable affective pattern in the sample, combining high positive affect, low negative affect, and consistent perceived social support.

Profile A, labelled High Negative Affect/Moderate-to-High Social Support, showed the highest level of negative affect (M = 20.12) and below-average positive affect (M = 18.38), while maintaining moderate-to-high levels of perceived social support: Significant Other (M = 6.30), Family (M = 5.82), and Friends (M = 5.86). This pattern suggests that, despite perceiving relatively strong social support, women in this profile reported substantial negative affect.

Profile C, labelled Low Social Support/Mixed Affective Pattern, was distinguished by the lowest levels of perceived social support across all three sources: Significant Other (M = 4.10), Family (M = 4.05), and Friends (M = 3.80). This profile also showed low positive affect (M = 17.88) and moderately elevated negative affect (M = 18.01). Thus, Profile C appears to reflect a more vulnerable affective and social support pattern, characterized by reduced positive affect, elevated negative affect, and limited perceived social support.

### 3.2. Cross-Analysis with Resilience and Displacement Status

**Figure 1:**
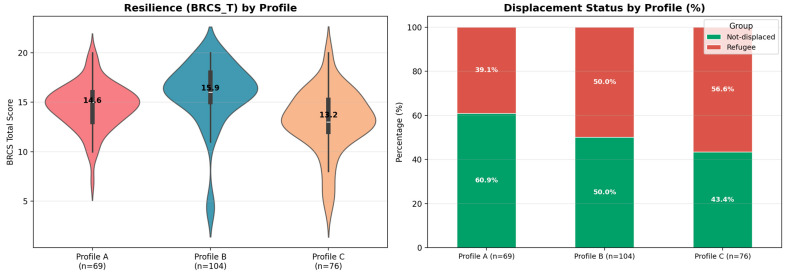
Resilience and displacement status across the identified profiles.

As described in Figure 1, resilience scores (BRCS-T) differed significantly across the three profiles, according to the Kruskal–Wallis test, H(2) = 50.13, *p* < 0.001, η^2^ = 0.196, indicating a large effect size.

Profile B showed the highest levels of resilience (Mdn = 16; M = 15.89, SD = 3.31), followed by Profile A (Mdn = 15; M = 14.62, SD = 2.26), whereas Profile C presented the lowest resilience scores (Mdn = 13; M = 13.16, SD = 3.14). Bonferroni-corrected post hoc Mann–Whitney U tests indicated significant differences between all profiles: Profile A versus Profile B (U = 2136, *p* < 0.001, r = 0.342), Profile A versus Profile C (U = 3418, *p* = 0.0015, r = 0.262), and Profile B versus Profile C (U = 6173, *p* < 0.001, r = 0.479). Effect sizes ranged from small-to-moderate to moderate magnitude, with the largest difference observed between Profiles B and C. Regarding displacement status, the proportion of non-displaced women and displaced women under temporary protection varied across profiles. Profile A included 60.9% non-displaced women and 39.1% displaced women under temporary protection, Profile B showed an equal distribution between groups (50.0% each), and Profile C included a higher proportion of displaced women under temporary protection (56.6%) than non-displaced women (43.4%). However, the association between displacement status and profile membership did not reach statistical significance, χ^2^(2) = 4.48, *p* = 0.107, Cramér’s V = 0.134, indicating a small effect size. Although displaced women under temporary protection appeared to be more represented in the most vulnerable profile, these differences should be interpreted cautiously, given the absence of statistically significant group differences.

## 4. Discussion

The present study explored emotional functioning, resilience, and social support among displaced and non-displaced Ukrainian women affected by the ongoing war. Contrary to assumptions that displacement necessarily leads to greater psychological maladjustment, displaced women showed slightly higher resilience scores on BRCS scores, although these differences did not reach statistical significance. At the same time, non-displaced women reported significantly higher levels of both positive and negative emotional experiences. This pattern suggests that individuals remaining in Ukraine may experience greater emotional intensity overall, potentially reflecting continuous exposure to war-related stressors and uncertainty. Such findings align with previous literature indicating that exposure to chronic adversity may coexist with adaptive coping processes and emotional complexity (Morimitsu & Akerkar, 2023).

Interestingly, displaced women under temporary protection reported lower levels of negative affect compared to non-displaced participants. This finding may reflect adaptive emotional regulation processes, posttraumatic growth, or shifts in personal priorities following displacement under temporary protection. In line with Morimitsu and Akerkar (2023) and Chowdhury et al. (2014), displaced populations may develop positive psychological changes despite severe hardships such as relocation, family separation, and economic instability. Experiences associated with displacement may be associated with higher levels of problem-solving abilities, adaptability, and self-reliance, characteristics that have been linked to resilience in previous research. Thus, the present results reinforce the idea that resilience should not be conceptualized merely as the absence of distress, but rather as a dynamic adaptation process occurring alongside emotional challenges.

The cluster analysis further revealed substantial heterogeneity in psychosocial functioning. Profile B, characterized by high positive affect, low negative affect, stronger social support, and the highest resilience scores, represented the most adaptive functioning pattern in the sample. In contrast, Profile C showed the lowest levels of perceived social support, elevated negative affect, and the lowest resilience scores, reflecting a pattern of combined emotional and social vulnerability. These findings are consistent with previous evidence emphasizing the protective role of social support and coping resources during periods of crisis and forced migration (Solà-Sales et al., 2021; Luthar et al., 2000). Particularly noteworthy was Profile A, which combined high levels of negative affect with moderate-to-high levels of perceived social support. This pattern suggests that social support may not function as a simple protective factor that directly reduces emotional distress under conditions of chronic adversity. In contexts of prolonged war exposure, individuals may continue to experience fear, uncertainty, grief, and ongoing stress even when they perceive their social relationships as available and supportive. From this perspective, women reporting higher levels of perceived social support also tended to report higher resilience, without necessarily reporting lower levels of negative emotional experiences. Consistent with this interpretation, women in Profile A displayed significantly higher resilience than those in the more vulnerable Profile C, despite reporting similarly elevated levels of negative affect. This finding aligns with contemporary conceptualizations of resilience, which define adaptation not as the absence of distress but as the capacity to maintain psychological functioning while facing ongoing emotional challenges. Taken together, these results suggest that emotional distress and psychosocial resources may coexist, particularly in populations exposed to prolonged conflict and uncertainty, highlighting the importance of distinguishing between emotional well-being and adaptive coping processes.

Literature on ageing and resilience suggests that older individuals may develop more effective emotional regulation strategies and coping mechanisms through accumulated life experiences, while younger ones may have more negative ones as a protective strategy (Reed & Carstensen, 2012; Kunzmann et al., 2000; Scharbert et al., 2024). In the present study, age emerged as a significant negative predictor of negative affect, whereas it was not significantly associated with resilience. This finding suggests that older women reported fewer negative emotional experiences, although they did not necessarily exhibit higher levels of resilient coping. Conversely, prolonged exposure to uncertainty and disrupted life trajectories may contribute to elevated emotional distress among those remaining in conflict zones (Miller & Rasmussen, 2010). Overall, the results underscore the complexity of emotional adaptation during war and displacement and suggest that vulnerability and resilience may coexist within the same populations.

Although displaced women under temporary protection appeared more represented in the most vulnerable profile, the association between displacement status and profile membership was not statistically significant. This finding highlights the heterogeneity of adaptation processes among displaced populations and cautions against overly deterministic interpretations of refugee status as inherently associated with psychological vulnerability. Similar to previous research, the present findings suggest that individual differences in resilience may depend not only on displacement itself, but also on broader psychosocial and contextual factors, including coping strategies, social relationships, and personal resources (Hooberman et al., 2010).

Several limitations should be acknowledged. The cross-sectional design precludes causal inferences, and the use of convenience sampling limits the generalizability of the findings. Another limitation concerns the stability of the k-means clustering solution. Although the three-cluster solution was selected based on the elbow method, silhouette coefficient, and psychological interpretability, no formal stability analyses were performed. Because k-means clustering may be sensitive to random initialization and outliers, the identified profiles should be considered exploratory and may partially reflect sample-specific clustering patterns. Future research should replicate these findings using larger samples and complementary validation procedures such as bootstrap resampling, split-half validation, or hierarchical clustering approaches. The comparison between displaced and non-displaced women should be interpreted with caution because the groups were not randomly assigned and may differ in several unmeasured characteristics associated with migration opportunities and resources. Furthermore, the inclusion of internally displaced women in future studies would help disentangle the effects of displacement from those related to migration and selection processes. In this regard, the initial group differences observed in resilience and affective experience should be interpreted with caution. After adjusting for age, displacement status was no longer significantly associated with resilience, positive affect, or negative affect, suggesting that the unadjusted differences between groups may have been partly attributable to age-related variation rather than displacement itself. In particular, age emerged as a significant predictor of negative affect, with older women reporting lower levels of negative emotional experiences. This finding is consistent with previous research indicating that older adults often exhibit greater emotional regulation and psychological adaptation when facing adversity.

In addition, the absence of longitudinal data prevents examination of how emotional functioning and resilience evolve over time in displaced and non-displaced populations. Future studies should explore longitudinal trajectories of adaptation, incorporating contextual variables such as duration of displacement, proximity to conflict exposure, and access to social and institutional support systems. Further research may also benefit from examining the potential influence of European protection policies, including the EU Council Directive 2001/55/CE, on psychological well-being and adjustment processes among displaced Ukrainian populations.

## 5. Conclusions

The results suggest that psychological vulnerability was not clearly associated with displacement status alone, as displaced women showed slightly higher resilience and lower negative affect than non-displaced women. However, displaced participants also reported fewer positive experiences, indicating that resilience may coexist with reduced emotional well-being. Cluster analyses identified three distinct psychosocial profiles, with the most adaptive profile characterized by high positive affect, low negative affect, stronger social support, and higher resilience. Although displaced women under temporary protection were more represented in the most vulnerable profile marked by low social support and emotional distress, this association was not statistically significant, highlighting the heterogeneity of adaptation processes among displaced populations.

## Figures and Tables

**Table 1:** Group Statistics.

Measure	Displaced	Mean	SD	*p* (*d*’)
BRCS	No	14.36	3.24	0.08 (−0.22)
	Yes	15.07	3.14	
SPANE_P	No	21.13	4.34	0.02 (0.30)
	Yes	19.87	4.17	
SPANE_N	No	17.91	4.05	0.001 (0.44)
	Yes	16.13	3.96	
Significant Other	No	5.80	1.27	0.017 (0.30)
	Yes	5.39	1.43	
Family	No	5.42	1.41	0.08 (0.02)
	Yes	5.39	1.41	
Friends	No	5.33	1.51	0.78 (0.03)
	Yes	5.28	1.27	

Note. M = mean, SD = Standard Deviation, SE = Standard Error. BRCS = Brief Resilient Coping Scale Total, SPANE_P = Scale of Positive and Negative Experience Positive, SPANE_N = Scale of Positive and Negative Experience Negative. *d*’= Cohen’s *d*’.

## Data Availability

Dataset available on request from the authors.

## References

[B1-behavsci-16-00988] Aloni R., Ben-Ari A. (2024). The role of comorbidity in Understanding traumatic sequelae among Ukrainian war refugees. Journal of Loss and Trauma.

[B2-behavsci-16-00988] Barzilay R., Moore T. M., Calkins M. E., Maliackel L., Jones J. D., Boyd R. C., Warrier V., Benton T. D., Oquendo M. A., Gur R. C., Gur R. E. (2021). Deconstructing the role of the exposome in youth suicidal ideation: Trauma, neighborhood environment, developmental and gender effects. Neurobiology of Stress.

[B3-behavsci-16-00988] Bonanno G. A. (2004). Loss, trauma, and human resilience: Have we underestimated the human capacity to thrive after extremely aversive events?. American Psychologist.

[B4-behavsci-16-00988] Bryan C. J., Griffith J. E., Pace B. T., Hinkson K., Bryan A. O., Clemans T. A., Imel Z. E. (2015). Combat exposure and risk for suicidal thoughts and behaviors among military personnel and veterans: A systematic review and meta-analysis. Suicide and Life-Threatening Behavior.

[B5-behavsci-16-00988] Chowdhury R., Sharot T., Wolfe T., Düzel E., Dolan R. J. (2014). Optimistic update bias increases in older age. Psychological Medicine.

[B6-behavsci-16-00988] Hooberman J., Rosenfeld B., Rasmussen A., Keller A. (2010). Resilience in trauma-exposed refugees: The moderating effect of coping style on resilience variables. American Journal of Orthopsychiatry.

[B7-behavsci-16-00988] Jankovic J., Bremner S., Bogic M., Lecic-Tosevski D., Ajdukovic D., Franciskovic T., Galeazzi G. M., Kucukalic A., Morina N., Popovski M., Schützwohl M., Priebe S. (2013). Trauma and suicidality in war affected communities. European Psychiatry.

[B8-behavsci-16-00988] Kovess-Masfety V., Keyes K., Karam E., Sabawoon A., Sarwari B. A. (2021). A national survey on depressive and anxiety disorders in Afghanistan: A highly traumatized population. BMC Psychiatry.

[B9-behavsci-16-00988] Kunzmann U., Little T. D., Smith J. (2000). Is age-related stability of subjective well-being a paradox? Cross-sectional and longitudinal evidence from the Berlin Aging Study. Psychology and Aging.

[B10-behavsci-16-00988] Luthar S. S., Cicchetti D., Becker B. (2000). The construct of resilience: A critical evaluation and guidelines for future work. Child Development.

[B11-behavsci-16-00988] Maley W. (2011). Afghanistan in 2010: Continuing governance challenges and faltering security. Asian Survey.

[B12-behavsci-16-00988] Miller K. E., Rasmussen A. (2010). War exposure, daily stressors, and mental health in conflict and post-conflict settings: Bridging the divide between trauma-focused and psychosocial frameworks. Social Science & Medicine.

[B13-behavsci-16-00988] Moret-Tatay C., Murphy M. (2022). Anxiety, resilience and local conditions: A cross-cultural investigation in the time of COVID-19. International Journal of Psychology.

[B14-behavsci-16-00988] Moret-Tatay C., Zharova I., Cloquell- Lozano A., Pérez-Bermejo M., Murphy M., Arteaga-Moreno F. (2025). Social support increases resilience and affect in non-displaced Ukrainians and refugees after a year of war. Psicothema.

[B15-behavsci-16-00988] Morimitsu R., Akerkar S. (2023). Rethinking forced migrants’ well-being: Lessons from Ukraine. Forced Migration Review.

[B16-behavsci-16-00988] Murphy M., Lami A., Moret-Tatay C. (2021). An Italian adaptation of the Brief Resilient Coping Scale (BRCS) and attitudes during the COVID-19 outbreak. Frontiers in Psychology.

[B17-behavsci-16-00988] Reed A. E., Carstensen L. L. (2012). The theory behind the age-related positivity effect. Frontiers in Psychology.

[B18-behavsci-16-00988] Rutter M. (2007). Resilience, competence, and coping. Child Abuse & Neglect.

[B19-behavsci-16-00988] Scharbert J., Humberg S., Kroencke L., Reiter T., Sakel S., Ter Horst J., Utesch K., Gosling S. D., Harari G., Matz S. C., Schoedel R., Stachl C., Aguilar N. M. A., Amante D., Aquino S. D., Bastias F., Bornamanesh A., Bracegirdle C., Campos L. A. M., Back M. D. (2024). Psychological well-being in Europe after the outbreak of war in Ukraine. Nature Communications.

[B20-behavsci-16-00988] Shalev A. Y., Gevonden M., Ratanatharathorn A., Laska E., Van Der Mei W. F., Qi W., Lowe S., Lai B. S., Bryant R. A., Delahanty D., Matsuoka Y. J., Olff M., Schnyder U., Seedat S., deRoon-Cassini T. A., Kessler R. C., Koenen K. C., International Consortium to Predict PTSD (2019). Estimating the risk of PTSD in recent trauma survivors: Results of the International Consortium to Predict PTSD (ICPP). World Psychiatry.

[B21-behavsci-16-00988] Shevlin M., Hyland P., Karatzias T. (2022). The psychological consequences of the Ukraine war: What we know, and what we have to learn. Acta Psychiatrica Scandinavica.

[B22-behavsci-16-00988] Sinclair V. G., Wallston K. A. (2004). The development and psychometric evaluation of the Brief Resilient Coping Scale. Assessment.

[B23-behavsci-16-00988] Singhal S. (2019). Early life shocks and mental health: The long-term effect of war in Vietnam. Journal of Development Economics.

[B24-behavsci-16-00988] Solà-Sales S., Pérez-González N., Van Hoey J., Iborra-Marmolejo I., Beneyto-Arrojo M. J., Moret-Tatay C. (2021). The role of resilience for migrants and refugees’ mental health in times of COVID-19. Healthcare.

[B25-behavsci-16-00988] Southwick S. M., Bonanno G. A., Masten A. S., Panter-Brick C., Yehuda R. (2014). Resilience definitions, theory, and challenges: Interdisciplinary perspectives. European Journal of Psychotraumatology.

[B26-behavsci-16-00988] Steel Z., Chey T., Silove D., Marnane C., Bryant R. A., Van Ommeren M. (2009). Association of torture and other potentially traumatic events with mental health outcomes among populations exposed to mass conflict and displacement: A systematic review and meta-analysis. JAMA.

[B27-behavsci-16-00988] UNHCR (2022). Displacement patterns, protection risks and needs of refugees from Ukraine.

[B28-behavsci-16-00988] UNHCR (2026). Ukraine emergency.

[B29-behavsci-16-00988] Vorobiova A., Schilling R. (2023). The impacts of war-induced stress on the health and physical activity of Ukrainians. ECSS Paris 2023—The 28th Annual Congress of the European College of Sport Science.

[B30-behavsci-16-00988] Wingo A. P., Wrenn G., Pelletier T., Gutman A. R., Bradley B., Ressler K. J. (2010). Moderating effects of resilience on depression in individuals with a history of childhood abuse or trauma exposure. Journal of Affective Disorders.

[B31-behavsci-16-00988] Zimet G. D., Dahlem N. W., Zimet S. G., Farley G. K. (1988). The multidimensional scale of perceived social support. Journal of Personality Assessment.

